# Mixture of easy trials enables transient and sustained perceptual improvements through priming and perceptual learning

**DOI:** 10.1038/s41598-017-06989-0

**Published:** 2017-08-07

**Authors:** Zhicheng Lin, Barbara Anne Dosher, Zhong-Lin Lu

**Affiliations:** 1 Department of Psychology, The Ohio State University, Columbus, USA; 2 Department of Cognitive Sciences, University of California, Irvine, USA

## Abstract

The sense of vision allows us to discriminate fine details across a wide range of tasks. How to improve this perceptual skill, particularly within a short training session, is of substantial interest. Emerging evidence suggests that mixing easy trials can quickly improve performance in hard trials, but it is equivocal whether the improvement is short-lived or long-lasting, and additionally what accounts for this improvement. Here, by tracking objective performance (accuracy) and subjective experience (ratings of target visibility and choice confidence) over time and in a large sample of participants, we demonstrate the coexistence of transient and sustained effects of mixing easy trials, which differ markedly in their timescales, in their effects on subjective awareness, and in individual differences. In particular, whereas the transient effect was found to be ubiquitous and manifested similarly across objective and subjective measures, the sustained effect was limited to a subset of participants with weak convergence from objective and subjective measures. These results indicate that mixture of easy trials enables two distinct, co-existing forms of rapid perceptual improvements in hard trials, as mediated by robust priming and fragile learning. Placing constraints on theory of brain plasticity, this finding may also have implications for alleviating visual deficits.

## Introduction

From acquiring a new word to memorizing a new face, a constant challenge to our perceptual system is to quickly learn novel perceptual information as we encounter them. What contributes to our success—and sometimes failure—in coping with this challenge? We hypothesize that exposure to multiple clear instances of the target information may be key in accelerating learning. This idea can be instantiated through easy trials in the laboratory, by exposing the visual system to highly-visible, target-defining objects. But it remains equivocal how the mixture of easy trials affects performance in hard trials. Earlier studies consistently show that after exposure to easy trials, our performance in the hard trials improves *permanently*—remaining at a substantially elevated level after the easy trials are no longer present (“perceptual insight” or “eureka”^1, 2^; or learned decision templates^3^). On the other hand, recent studies demonstrate that exposure to easy trials leads only to *short-lived* improvement—the improvement vanishes when the easy trials are removed (“priming of awareness”^4, 5^).

These contradictory observations present a roadblock to our understanding of visual learning^6^, raising the fundamental question of whether and how easy trials may enable genuine fast learning—learning that takes place within a session as opposed to days or longer—in the first place. We therefore set out to reconcile these findings and to shed light on the mechanism that enables perceptual improvements from mixing easy trials. We do so by devising a large-scale study that measured both objective performance (accuracy) and subjective experience (visibility and confidence ratings). This design allowed us to evaluate both objective and subject effects from the mixture of easy trials and furthermore to characterize individual differences.

## Results

**Figure 1:**
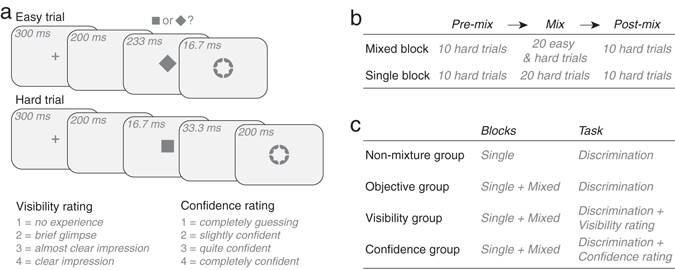
Experimental design. (**a**) On each trial, a square or a diamond was presented either for 233 ms before it was masked, making it easy to recognize (an easy trial); or briefly for 16.7 ms and so it was hard to recognize (a hard trial). (**b**) The presence or absence of easy trials defined two types of blocks: a mixed block that consisted of 10 hard trials, 20 alternating easy and hard trials, and 10 hard trials; a single block consisting of 40 hard trials. (**c**) There were four groups (*n* = 28 each) based on the mixture of easy trials and the task. Whereas the non-mixture group trained in 28 single blocks, the objective, visibility, and confidence groups went through 28 interleaving single and mixed blocks (half started with a single block; the other half, a mixed block).

One hundred and twelve participants were recruited. Figure 1a shows the structure of easy and hard trials. In each trial a square or a diamond was presented for one of two durations before it was masked: 233 ms in easy trials, 16.7 ms in hard trials. The objective task was to discriminate whether the target was a square or a diamond; the subjective task, to rate either target visibility or choice confidence. Based on the presence or absence of 1) easy trials and 2) a subjective task, participants were separated into 4 groups (*n* = 28 each): a non-mixture (i.e., no easy trials), identification-task-only group (hereafter, *non-mixture* group); and three mixture groups—an identification-task-only group; an identification plus visibility rating group; an identification plus confidence rating group (hereafter, *objective*, *visibility* and *confidence* groups). First, the non-mixture group went through 28 blocks of hard trials without easy trials (single blocks); the three mixture groups went through 28 interleaving single and mixed blocks (Fig. 1b). A mixed block consisted of 10 hard trials (pre-mix), 20 alternating easy and hard trials (mix), and then 10 hard trials (post-mix). Importantly, interleaving mixed and single blocks allowed us to isolate and track sustained and transient effects from easy trials: sustained changes as indexed by changes in performance *across* single blocks; transient changes as indexed by changes in performance *within* a given mixed block. Second, the non-mixture group and the objective group completed only the identification task; the visibility group and the confidence group did the same experiment as the objective group, but additionally were asked to indicate, on a trial-by-trial basis, their subjective experience regarding target visibility or choice confidence on a 4-point scale (Fig. 1a).

**Figure 2:**
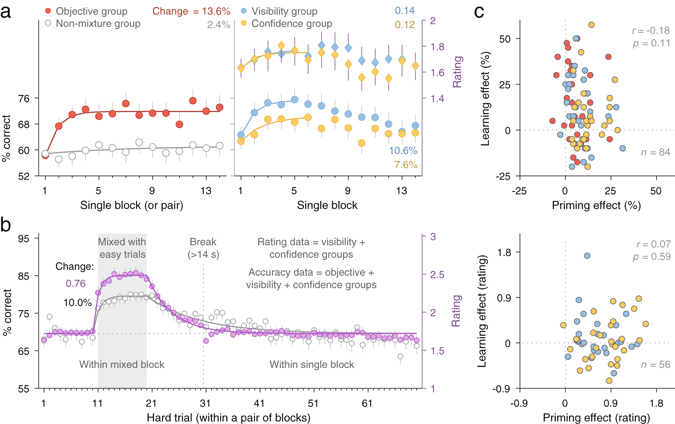
Sustained and transient effects from the mixture of easy trials. (**a**) Sustained perceptual improvements in single blocks were evident in the three mixture groups (relative to the non-mixture group). The objective, visibility, and confidence groups went through 14 pairs of mixed and single blocks; the non-mixture group, 14 pairs of single blocks (28 blocks in total). Each data point represents the average of a single block (two single blocks for the non-mixture group). Connecting lines were fitted based on an exponential learning model (see text). *B* is a parameter in the model, representing the magnitude of learning. Error bars (one-sided) are standard errors of the mean. (**b**) Trial-by-trial transient improvements were shown in a pair of mixed–single blocks. The mixed–single block pair was constructed by averaging across 14 pairs of blocks (for the participants who started with a single block) or 13 pairs (for those starting with a mixed block, as the first mixed block and the last single block were excluded). Hard trials were numbered based on their order within each pair of mixed–single blocks, excluding the easy trials. The horizontal dash line represents the mean performance in the first 10 hard trials (aligning accuracy on the left axis and rating on the right axis). The connecting lines were fitted based on an exponential priming–decay model (see text). (**c**) Transient (priming) and sustained (learning) improvements did not correlate with each other, either in accuracy or in rating.

Our first objective was to ascertain that any sustained changes in single blocks were due to the mixture of easy trials in mixed blocks rather than other factors (such as mere practice). Therefore we tracked and compared performance in single blocks between the objective group and the non-mixture group, which differed only in the mixture of easy trials. As Fig. 2a (left) shows, the mixture of easy trials bestowed a clear performance advantage that emerged quickly and persisted to the end of the session (main effect of group: *F*(1, 54) = 81.38, *p* < 0.001, ${\eta }_{p}^{2}$ = 0.60; main effect of block pairs: *F*(13, 702) = 15.21, *p* < 0.001, ${\eta }_{p}^{2}$ = 0.22; interaction: *F*(13, 702) = 9.15, *p* < 0.001, ${\eta }_{p}^{2}$ = 0.14). To estimate and compare the sustained effect, we fitted the data presented in Fig. 2a using the formula, *A* + *B* (1 − *e* ^−(*t−1*)/*T*^), with 3 parameters: *A*, initial performance; *B*, maximum amount of learning; and *T*, the time constant for learning (see Section “Model method” in Supplementary Materials). Although in both groups this learning model outperformed a model that assumed no learning, the learning effect (*B*) was much more pronounced in the objective group (mean ± one standard deviation, *SD*: 13.6% ± 3.5%) than in the non-mixed group (2.4% ± 3.1%). The estimated learning rating (*T*) revealed that it took about 2.6 (±1.2) blocks for the objective group to reach 95% of maximum learning (see Supplementary Table S1 and Supplementary Section “Model statistics”).

For the two rating groups, sustained changes were also evident, emerging within the first few single blocks (Fig. 2a, right). Performance then tapered off. Performance deterioration within a single testing session is not uncommon^7^, which can be attributed to sensory adaptation or fatigue. Because sensory adaptation depends on the number of trials and the number of trials was the same for the two non-rating groups and for the two rating groups, their differences were likely due to a difference in the fatigue effect. Fatigue likely arose from the demanding nature of the dual task and the longer time required for completing the study (about 1.5 hours; cf., one hour for the two objective-task-only groups). Learning therefore was modelled prior to performance decline (cut off liberally at the block with the highest accuracy; hence 5^th^ block in the visibility group, 6^th^ block in the confidence group). The estimated learning effect (*B*) was 10.6% ± 3.9% for the visibility group (in ratings: 0.14 ± 0.20) and 7.6% ± 4.0% for the confidence group (in ratings: 0.12 ± 0.11), higher than the non-mixture group (see Supplementary Table S1 and Supplementary Section “Model statistics”).

To probe transient effects, we tracked trial-by-trial changes in performance within a mixed block, as compared with changes within a single block. As Fig. 2b shows, in both accuracy and ratings, mixture of easy trials led to rapid improvement in the mixed block, which gradually decayed when easy trials were removed. Going into the single block, the effect all but completely disappeared. This finding indicates that changes in performance across single blocks—shown in Fig. 2a and detailed above—must be due to sustained changes rather than to some residual transient effects from mixed blocks. Moreover, that performance did not improve in the middle of trials within the single block demonstrates that the transient change that did occur in the mixed block must be due to the mixture of easy trials rather than to some trial order effect. Separately examining the transient effect in early and late session further revealed that the transient effect was comparable between the first 5 block pairs, when sustained improvements rapidly took place in single blocks, and the remaining block pairs, when performance already stabilized in single blocks (see Supplementary Fig. S1). Together these results demonstrate that the transient effect can coexist with, but is also dissociable from, the sustained effect.

We fitted the transient effect with a priming–decay model, *A* + *B* (1 − *e* ^−(*t−1*)/*T1*^), when *t* < 11 (pre-mix and mix trials); *A* + *B* × *e* ^−(*t* − 11)/*T2*^, when *t* ≥ 11 (post-mix and single-block trials; *A*, initial performance; *B*, maximum amount of priming; *T1*, time constant of priming; and *T2*, time constant of decay). The priming effect (*B*) was comparable between the visibility and confidence groups (in accuracy, 9.4% vs. 14.0%; in ratings, 0.67 vs. 0.86; see Supplementary Table S2). On average, it took about 3.3 (±1.2) trials and 2.7 (±0.4) trials to reach 95% of maximum priming in accuracy and ratings, respectively—much quicker than that in the sustained effect (cf. 2.6 blocks in the objective group). The effect declined by 95% when the trial number in Fig. 2b reached trial 49 ± 4 for accuracy and trial 34 ± 2 for ratings, well before the end of the single block.

The common perceptual improvement parameter, *B*, allowed us to compare the transient effect (priming) and the sustained effect (learning). With regard to improvements in accuracy, the estimated magnitude of the priming effect was 10.0% (±1.0%, Fig. 2b), comparable to that of the learning effect (13.6%, 10.6%, 7.6%; Fig. 2a). However, with regards to improvements in ratings, the priming effect was 0.76 (±0.05), 5 times as large as the learning effect (0.14, 0.12; see section “Model statistics” in Supplementary Materials). To confirm, we evaluated learning in a different way, by looking at changes across *mixed* (rather than single) blocks. Supplementary Fig. S2 showed block-by-block accuracy and ratings in the premix, mix, and postmix segments of hard trials. Here too the magnitude of the learning effect in accuracy (9.6%, 6.1%) was comparable to that of the priming effect, but the learning effect in ratings (0.15) was one fifth of the priming effect (see Supplementary Table S3). These results demonstrate a clear dissociation between priming and learning in their accuracy and rating effects.

Do sustained and transient perceptual improvements reflect distinct mechanisms involved in the mixture effects, or do they arise from a single underlying mechanism that manifests at different timescales? If the two effects are enabled by a common mechanism, one would expect those participants with a larger transient effect to also show a larger sustained effect. To test this hypothesis, we examined Pearson’s correlation between transient and sustained effects (Fig. 2c). Their correlation was slightly negative in terms of accuracy (*r* = −0.18) and slightly positive in ratings (*r* = 0.07), and neither were statistically significant (*p* = 0.107 and *p* = 0.588). In other words, the magnitude of the transient effect did not predict the magnitude of the sustained effect. These results therefore suggest that transient and sustained improvements reflect distinct mechanisms in the mixture effects.

Therefore, tracking changes in performance across single blocks revealed a sustained learning effect that plateaued in 3 blocks (Fig. 2a). At the same time, tracking changes in performance within mixed blocks unveiled a transient priming effect that took place after the mixture of just one easy trial, with the effect reaching plateau after exposure to 3 easy trials (Fig. 2b). Priming and learning induced comparable degrees of improvement in accuracy, but priming exerted 5 times as large an improvement in ratings as learning did, providing strong evidence for a dissociation between objective and subjective effects. Moreover, priming and learning did not correlate with each other, indicating distinct mechanisms that are involved in the mixture effects from easy trials. Together, these results reveal that priming and learning underlie rapid perceptual improvements from easy trials, and the two effects (1) co-exist with each other but manifest at different timescales and are dissociable, (2) exert comparable effects on accuracy but show sharply different effects on ratings, and (3) reflect distinct mechanisms.

Thus far, as in traditional perceptual learning studies, we have focused on group averages. Unlike most prior studies, however, two features of the current study—relatively large sample, conjoint measures of accuracy and ratings—afforded us to address individual differences as related to objective and subjective measures. Besides their theoretical importance in perception and learning, these issues are also central in potential clinical applications, because individual differences determine the robustness and efficacy of interventions and because subjective measures provide a critical index of our perception that do not always agree with objective measures.

**Figure 3:**
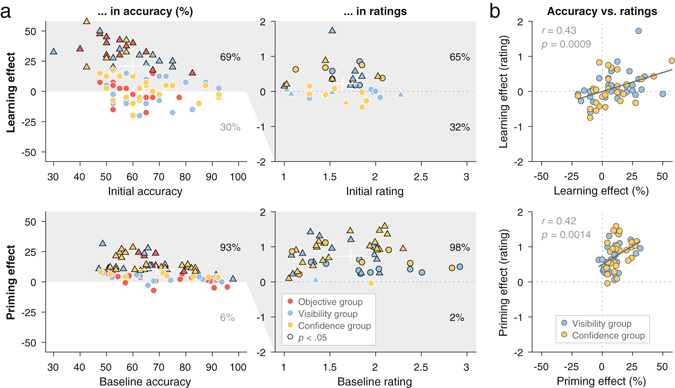
Distinct contributions of learning and priming to rapid perceptual enhancement in hard trials. (**a**) The learning effect in accuracy (top panels, *y* axis)—the difference in identification accuracy between the single block at peak performance and the first single block (i.e., initial accuracy, *x* axis)—was positive in 69% of participants (left), and only a subset of them showed corresponding improvements in visibility or confidence ratings (right). On the other hand, the priming effect in accuracy (bottom panels, *y* axis)—the difference in hard-trial accuracy between the mix segment and the pre-mix segment (i.e., baseline, *x* axis)—was positive in 93% of participants (left), and most of them showed corresponding improvements in visibility or confidence ratings (right). Percentages represent the proportions of participants showing positive or negative effects; there were three mixture groups for accuracy and two rating groups for ratings. Each dot or triangle represents an individual participant (triangle denotes statistically significant accuracy effect at the individual level, *p* < 0.05); black contour denotes statistically significant effect at the individual level, *p* < 0.05, one-tailed (white contour, non-significant effect); center of the white bar, mean of all the individuals within the highlighted (gray) region; full length of the white bar, a standard deviation. (**b**) Accuracy effects and rating effects were strongly correlated with each other, with comparable magnitudes in learning and in priming. The solid line represents linear regression fitting; it was shallower in learning (top) than in priming (bottom), but the difference was not statistically significant (*F*(1, 108) = 2.03, *p* = 0.157).

To examine whether and how sustained learning and transient priming differed in individual differences, we calculated each individual participant’s learning and priming effects: the learning effect as the performance difference between the first single block and, to minimize the impact of fatigue, the single block of the highest identification accuracy (i.e., blocks 12, 5, and 6 for the objective, visibility, and confidence group, respectively); the priming effect as the performance difference between pre-mix and mix hard-trials (averaged across all the mixed blocks). As Fig. 3a shows, for learning, 58 participants showed improvements in identification accuracy (i.e., positive learning effects in accuracy), and only 65% of them showed corresponding improvements in ratings (i.e., positive learning effects in ratings). In contrast, for priming, 78 participants showed improvements in identification accuracy, and 98% of them also showed corresponding improvements in ratings, a much higher ratio than that in learning (*X* ^*2*^ (1, *N* = 91) = 16.21, *p* < 0.001).

Obviously, some positive effects did not reach statistical significance at the individual level (as marked by white contours in Fig. 3a), which may reflect either noise or true effects that went undetected because of low statistical power. Aggregating across individuals improves power, providing a means to separate noise from true effects. In learning, the group accuracy effect from individuals with non-significant effects still failed to reach significance (*t*(53) = 0.01, *p* = 0.99), evidence consistent with noise. In priming, however, the group accuracy effect from individuals with non-significant effects surpassed significance (*t*(41) = 5.82, *p* < 0.001), providing evidence for the presence of some true effect that went undetected at individual levels. Examining only individuals who showed statistically significant improvements in identification accuracy (42 of them in priming, 30 in learning; as marked by black contours in Fig. 3a) revealed that more of them also showed significant improvements in rating for priming than for learning (97% vs. 63%*X* ^2^ (1, *N* = 51) = 8.40, *p* = 0.004). These results suggest that the priming effect was ubiquitous and manifested similarly across objective and subjective measures, whereas the learning effect was limited to only a subset of participants with weak convergence from objective and subjective measures.

What might explain the differences between priming and learning in individual differences? It was not the case that objective and subjective measures were more dissociable in learning than in priming, because Pearson’s correlation between accuracy and rating effects was comparable in learning (*r* = 0.43, *p* < 0.001) and in priming (*r* = 0.42, *p* = 0.001; Fisher’s *z* = 0.10, *p* = 0.924; Fig. 3b). It also was not that the priming effect was stronger than the learning effect; in contrast, for participants from the two rating groups whose accuracy effect was positive, the mean accuracy effect was lower in priming than in learning (11.2% ± 7.8% vs. 18.4% ± 13.7%; *t*(89) = −3.17, *p* = 0.002, Hedges’ *g* = −0.67).

**Figure 4:**
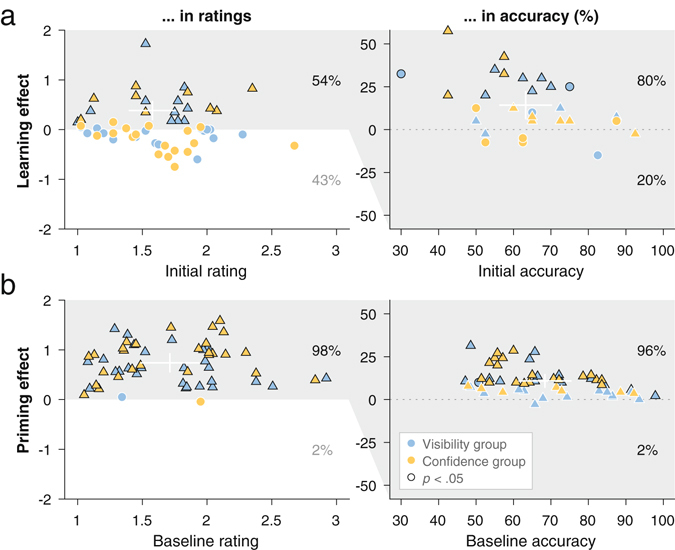
Distinct contributions of learning and priming to rapid perceptual enhancement in hard trials, as revealed by conditioning accuracy effects on positive rating effects. (**a**) Learning was positive in terms of *ratings* in 54% of participants (left), and only a subset of them showed a corresponding *accuracy* effect (right). (**b**) Priming was positive in terms of *ratings* in 98% of participants (left), and most of them showed a corresponding *accuracy* effect (right). Note that with the same data set the order of conditioning (testing accuracy effects based on positive rating effects) was the reverse of Fig. 3. Percentages represent the proportions of participants showing positive or negative effects. Each dot or triangle represents an individual participant (triangle denotes statistically significant rating effect at the individual level, *p* < 0.05, one-tailed); black contour denotes statistically significant effect at the individual level, *p* < 0.05 (white contour, non-significant effect); center of the white bar, mean of the individuals within the highlighted (gray) region; full length of the white bar, a standard deviation.

Instead, it appears that the learning effect was noisier than the priming effect. To corroborate this notion, we went beyond looking at rating effects based on *positive accuracy* effects (as in Fig. 3a), and did the reverse, by looking at accuracy effects based on *positive rating* effects. Figure 4 shows that for learning, 30 participants showed improvements in ratings, and 80% of them showed corresponding improvements in accuracy. For priming, 55 participants showed improvements in ratings, and 96% of them showed corresponding improvements in accuracy, a proportion significantly higher than that in learning (*X* ^*2*^ (1, *N* = 85) = 4.33, *p* = 0.038). The superior robustness in priming relative to learning could not be attributed to statistical reasons such as a difference in the number of trials (see Supplementary Materials, Section “A statistical account of differing individual differences between priming and learning” and Figs S4 to S6).

## Discussion

Our study provides the first demonstration that easy trials rapidly and simultaneously induce transient and sustained perceptual improvements on performance in hard trials. The transient improvement is characterized by a rapid but short-lived improvement following the mixture of easy trials within mixed blocks—an effect that is abolished going into the single block (Fig. 2b). The sustained improvement is characterized by a long-lived improvement across single blocks as a result of mixing easy trials in mixed blocks (Fig. 2a). Persistent improvements across single blocks in the objective group (relative to the control, non-mixture group) indicate discrimination learning—that is, successful short-term consolidation. Although testing across multiple days is needed to evaluate it in longer timescales, the sustained effect identified here lasted much longer than the within-block transient effect, and sustained across at least 12 single blocks of the current study.

These results help contextualize previous claims regarding the effect of mixing easy trials on hard trials: insight or eureka like improvement^1–3^ and priming like improvement^4, 5^. Whereas research on perceptual insight and eureka observed one-shot, long-lasting perceptual improvements, research on priming of awareness found stimuli-dependent, transient improvements. These findings invite one to interpret the different effects as enabled by some perhaps common mechanisms that manifest under different circumstances (e.g., in different stages of perceptual improvements). Such an interpretation would bear a conceptual resemblance to the notion that perceptual learning can proceed in two sequential stages of improvement: an early stage characterized by fast, rapidly saturating improvement^8^, and a late stage with slow learning^9^. The current findings, however, provide evidence against this interpretation, suggesting instead that transient and sustained improvements reflect two distinct but coexisting forms of plasticity: priming at the trial-by-trial scale; fast learning at the block-by-block scale.

By exposing the two coexisting scales of neural plasticity, our findings also suggest that mixture effects that have been previously assumed to reflect learning might include effects from priming. For example, in a study investigating how mixture of easy trials helps perceptual learning in hard trials, Liu *et al*.^3^ tracked performance in blocks that mixed easy and hard trials across 6 days. The authors found that performance in the hard trials (as measured by contrast threshold) improved in the course of training without performance feedback, an effect that was assumed to reflect learning. This assumption was largely reasonable, considering that mixture effects had been thought to reflect either sustained or transient improvement and that in this case the effect spanned over several blocks (days). The current study, however, reveals that the sustained effect at least partially reflected priming, because by mixing easy and hard trials within the same block, improvement in hard trials potentially reflected a trial-by-trial scale of improvement.

While our findings provide evidence for the existence of fast learning as induced by easy trials, the substantial individual differences in rapid sustained improvements are not anticipated in previous research on perceptual insight and eureka. In studies reporting insight or eureka, most if not all observers were reported to show rapid, large improvements^1, 2^. Indeed, the very term insight or eureka implies a *sudden* realization of something not understood before. This description is apt in some limited cases, such as in perceptual organization involving two-tone or fragmented images, where just one easy trial can quickly disambiguate and permanently improve our perception; learning in such cases involves complex images that must be taught in a case-by-case fashion. When the identity and the particular exemplars are already known to the observer (square and diamond in our case), improved identification of the relevant sensory evidence prior to the mask may be involved in learning. Furthermore, despite going through a pre-training session of 6 easy trials and 6 hard trials, performance for the non-mixture group stayed low (~60%) throughout the training session. That is, there is no “eureka” effect when the number of easy trials is small, implying a *gradual* learning process.

According to memory research, the individual differences observed here indicate the involvement of short-term consolidation in fast learning. Although the target in an easy trial can help fine-tune the neural code for the target signal (i.e., the target template), to prevent the template from fading after acquisition, it is essential to rapidly consolidate it into a more permanent representation, such as through fast synaptic consolidation^10^. Multiple exposures to easy trials help contribute to successful short-term consolidation, after which improvement can persistent in the absence of easy trials. This hypothesis is consistent with an influential computational theory^11^, which proposes that perceptual improvement entails an improvement in the likelihood estimation—the probability of the input given each possible state of the scene (in our case, square vs. diamond). Accordingly, perceptual improvement can be attributed to an improvement in the internal generative model^12^. One possibility is that the target template is being rapidly consolidated and optimized by easy trials through re-weighting^13–16^: an increase in the weights of target-related information and a decrease in the weights of target-unrelated information.

Unlike block-by-block fast learning, the trial-by-trial priming effect is highly dependent on episodic experience of easy trials. That is, the improvement is stimulus-driven, triggered by the target in the easy trial^4, 5^. Each exposure helps activate task-relevant neurons in visual cortex^17^, temporally improving the formation of target templates. Such perceptual improvement can be implemented by a matched filter algorithm, by correlating the temporally enhanced neural target template with the current sensory input to detect the presence of the target in the stimuli. The output, effectively, is a measure of the similarity between the thing we’re looking at (sensory input) and the thing we’re looking for (target templates).

Another important difference between priming and fast learning is that, while priming and learning improved accuracy to a similar degree, priming increased ratings—both target visibility and choice confidence—to a much larger extent than learning did, by a factor of five. This difference is dramatic and unexpected. One possibility is that since the priming observed here reflects an improvement that occurs after the mixture of just one easy trial, the change in perceptual quality is immediate and conspicuous. One cannot help but notice the sudden enhancement in perception—the hard trials appear to be not as hard as those in single blocks. On the other hand, since fast learning occurs over several blocks, the change in perception is less immediate and less dramatic. One may not be fully aware of an improvement in performance. Subjective awareness, in other words, is uniquely sensitive to the rate of change. In this sense, objective gain in performance is time discounted with respect to its reflection in awareness: the same improvement that occurs right now looms larger than that occurs later.

How might we explain the key findings here at the neurobiological level, that exposure to easy trials enables two distinct effects across different timescales? It is widely recognized that the functional units in the brain—cell assemblies—are composed of an interconnected group of neurons in a neural network as a result of frequent simultaneous neuronal activity^18, 19^. This scheme suggests that perceptual improvement reflects the strengthening of cell assemblies. Specifically, the presentation of a clear target elicits transient neuronal responses in a cell assembly responsible for its perception. When a subsequent hard trial is presented, the improved connection strength trained by clear targets promotes identification of the degraded target, and perhaps increases the effective sensory quality for the degraded target. This transient boost in sensory quality can contribute to perceptual enhancement—explaining trial-by-trial priming. On the other hand, when the memory trace from the clear target is re-activated, stabilized, and strengthened by continuous exposures to clear targets^20, 21^, as a results of short-term synaptic plasticity between cells^22^, it undergoes rapid synaptic consolidation, transitioning into a more stable and effective cell assembly^23^. Although necessarily speculative, this account indicates that when there is a sustained effect from template enhancement (i.e., fast learning), one can also observe a transient effect (i.e., priming).

In sum, our results provide the first demonstration that easy trials rapidly and simultaneously induce transient and sustained perceptual improvements. These two effects do not arise from a common underlying mechanism that manifests under different conditions, but instead reflect two different mechanisms that have distinct but coexisting scales of neural plasticity: priming at the trial-by-trial scale; fast learning at the block-by-block scale. A consequence of the difference in timescale is that priming exerts a much larger effect in awareness than learning does even when they show comparable effects on accuracy, indicating that awareness is highly sensitive to the rate of change. Perceptual gain, in other words, is time-discounted with regards to its reflection on awareness. The finding that objective and subjective measures dovetailed with each other in priming but showed weak convergence in learning reveals the fragile, noisy nature of fast learning, which is also less ubiquitous than priming across participants. By improving object templates in the visual system, easy trials help us see better, and fast. Perceptual enhancement from easy trials—or, *seeing to see*—offers a promising alternative to traditional slow perceptual learning to help improve perception in normal vision and potentially in low vision as well.

## Methods

### Participants and apparatus

One hundred and twelve students with normal or corrected-to-normal vision participated in the study. The study was approved by the Ohio State University Institutional Review Board and was performed in accordance with the relevant guidelines and regulations; informed consent was obtained from all participants.

Participants were separated into 4 groups (*n* = 28 each): a *non-mixture*, control group and 3 *mixture* groups. For the non-mixture group (21 females, 7 males; average age = 20.1), the experiment consisted solely of hard trials (Fig. 1a), and the task was to discriminate the target shape in each trial (as detailed in the next section). The 3 mixture groups went through mixture of easy and hard trials, with differing task requirements. One group was asked to perform, as the control group, the shape identification task (the *objective* mixture group; 18 females, 10 males; average age = 20.3); the other two groups were additionally asked to rate their experience on each trial: either regarding target visibility (the *visibility* mixture group; 13 females, 15 males; average age = 19.6; Fig. 1a) or regarding choice confidence (the *confidence* mixture group; 14 females, 14 males; average age = 20.9; Fig. 1a). We aimed to have at least the same sample size as a previous study using a similar procedure n = 16 in their Experiment 1^4^.

Participants were tested in one of two rooms (with equal proportion for each group). In one room, the stimuli were presented on a 19-inch CRT monitor (Dell M993S at 60 Hz and 1024 × 768 pixels; peak luminance: 20.0 cd/m^2^; black level: about 0.2 cd/m^2^), with a viewing distance of approximately 94 cm. In the other room, the stimuli were presented on a 19-inch CRT monitor (Dell P992 at 60 Hz and 1024 × 768 pixels; peak luminance: 95.2 cd/m^2^; black level: about 0.2 cd/m^2^), with a viewing distance of approximately 50 cm. A chin rest was used to stabilize head position. Lighting came only from the computer and the monitor.

### Procedure and design

Each experiment began with a 2-min, computerized fixation training session. A target dot was presented at the center of the screen on a square image. The square image was composed of black-and-white patches flickering in counterphase—each pixel alternated between black and white across frames. The task was to fixate on the central dot. Feedback on fixation stability was provided in real time: whenever the fixation deviated from the dot, it would instantly induce a flash of visual noise on the square image^24^.

After completing fixation training, participants proceeded to the main experiment. Figure 1a illustrates the procedure of each trial. A fixation cross (length = 0.23°; width = 0.08°) first appeared for 300 ms in the center of a black background, followed by a 200-ms blank screen (black). A target shape then appeared, equally likely to be a square (size = 0.93° × 0.93°) or a diamond (45° of rotation from the square), and stayed on the screen for either 233 ms (making it easy to recognize—referred to as an *easy trial*) or 16.7 ms (hard to recognize—a *hard trial*). The offset of the target was immediately followed by a 16.7-ms annulus (size = 1.54° × 1.54°) in easy trials, but was followed by a 33.3-ms blank screen first and then by a 200-ms annulus in hard trials. The annulus, by sharing the contour of the square and diamond, served as a metacontrast mask. The fixation, target, and annulus were at peak luminance.

The task was to judge whether the target was a square or a diamond, by clicking the left or right button on a mouse with the right hand. Afterward, participants in the visibility and confidence mixture groups—but not the objective mixture group or the non-mixture group—were further asked to rate their subjective experience on that trial, focusing on either the visibility of the target (for the visibility group) or their confidence in the shape judgement (for the confidence group). Four options were presented for visibility ratings: 1 = *no experience* (of the target; i.e., did not see it at all); 2 = *brief glimpse* (of the target but could not recognize what it was); 3 = *almost clear impression* (of the target, though not 100% sure what it was); 4 = *clear impression* (of the target; i.e., 100% sure what it was). Participants were instructed to rate the target rather than the mask (which was always highly visible). For confidence rating, the four options were: 1 = *completely guessing*; 2 = *slightly confident*; 3 = *quite confident*; 4 = *completely confident*. In both cases, the four choices were presented around the center of screen in four rows, and participants were asked to select their choice with their left hand on the keyboard. Specifically, one of the rows, selected in random for each trial, was highlighted by default; by pressing the *up* or *down* arrow key, the row above or below the currently highlighted one would instead become highlighted, and pressing the *right* arrow would confirm the highlighted row as the choice. No feedback was provided for performance or rating.

The experiment consisted of 28 blocks, 40 trials each. For the non-mixture group, the 28 blocks were made up of hard trials, without the mixture of easy trials—referred to as *single* blocks. For the mixture groups, every other block (odd blocks for half of the participants, even blocks for the other half) was replaced by a block that mixed easy and hard trials—referred to as a *mixed* block. It consisted of 10 hard trials, 20 alternating easy and hard trials, and 10 hard trials (in that order); the hard trials in the three segments were respectively referred to as the *pre-mix*, *mix*, and *post-mix* trials (Fig. 1b). A mandatory 15 s must lapse before participants could proceed to the next block (by pressing the space bar). All participants in the four groups practiced 12 alternating easy and hard trials at the beginning of the experiment.

### Data analysis

Anticipatory responses—reaction time under 100 ms for target identification—were rare (*M* = 0.09%, *SD* = 0.22%, range = [0 1.34%]). To allow for trial-by-trial analyses, trials with anticipatory responses were not excluded. The sphericity assumption of ANOVAs was tested with the Mauchly’s test of sphericity; when the assumption was violated, Greenhouse–Geisser correction was applied. Statistical significance at the individual level was evaluated using either Binomial tests (for accuracy) or Mann–Whitney U tests (for ratings). Correlation coefficients were compared using Fisher’s *z* transformation.

## Electronic supplementary material

Supplementary Materials
