# Supplementary material for: Mixture of easy trials enables transient and sustained perceptual improvements through priming and perceptual learning
Source: Sci Rep. 2017 Aug 7;7:7421. doi: 10.1038/s41598-017-06989-0 (PMC5547163; doi:10.1038/s41598-017-06989-0)
Supplement: Supplementary file 1 — Supplementary Materials [file 41598_2017_6989_MOESM1_ESM.pdf]

Supplementary Materials

Mixture of easy trials enables transient and sustained perceptual improvements through priming and perceptual learning

Zhicheng Lin, Barbara Anne Dosher, Zhong-Lin Lu

Model method. This section details three aspects of modelling: evaluating the quality of fit of a model; estimating the variability of model parameter; and comparing models.

First, how well a model explained the data was evaluated using the least-squares method, based on r-squared:

$$r^2 = 1 - \text{SSE from the model/SSE based on the mean value},$$

in which SSE is the sum of squared errors—error is the difference between the predicted and actual values.

Second, the variability of model parameters was estimated based on 1000 repetitions of bootstrap resampling; each resampled data set was resampled with replacement from the original data, maintaining the original sample size.

Third, in selecting models, nested models were constructed in which one model had fewer parameters than the other. For example, a full or saturated model would contain all the parameters, whereas a restricted or reduced model would contain fewer parameters. Nested models were compared by testing whether the more complex model significantly reduced the SSE for the extra parameter(s), using a F test:

$$F = (\text{SSE}_{restricted} - \text{SSE}_{full})/(p - k)/(\text{SSE}_{full}/(n - p - 1)),$$

in which, $\text{SSE}_{restricted}$ and SSE_{full} are the SSE from the restricted and full models, respectively; p and k are the number of parameters in the full and restricted models, respectively; n is the number of predicted data points.

This approach was also used to test whether the estimated value of a parameter differed between two groups or between two conditions within the same group. For example, to compare a parameter between two groups, two nested models were constructed: a full model that assumed two separate sets of parameters for the two groups; a restricted model in which the concerned parameter was assumed to be the same for the two groups (i.e., one fewer parameter).

Model statistics. The presence or absence of perceptual improvement was evaluated by comparing, in the case of learning, a learning model with a no learning model; and in the case of priming, a priming–decay model with a no priming (decay) model.

Specifically, the learning model is given by:

$$A + B\,(1 - e^{-(t-1)/T}),$$

in which, A, initial performance; B, maximum amount of learning; T, the time constant for learning, which determines the shape of the curve (i.e., the learning rate).

The priming–decay model is given by:

$A + B\,(1 - e^{-(t-1)/T1})$, when $t < 11$ (pre-mix and mix trials);
$A + B \times e^{-(t-11)/T2}$, when $t \geq 11$ (post-mix and single-block trials),

in which, *A*, initial performance; *B*, maximum amount of priming; *T1*, time constant of priming, which determines the rising rate; *T2*, time constant of decay, which determines the decline rate.

The no learning model and the no priming (decay) model assumed $B = 0$. Note that, in evaluating perceptual improvement, the initial performance *A* was fitted using the performance level from block pair 1 ($t = 1$)—*A* was not a free parameter. The results from these comparisons are provided in Tables S1, S2, and S3.

To compare perceptual improvements between two groups, the full model assumed two different *B* values for the two group, whereas the restricted model assumed the same *B* value for both groups. Compared with the non-mixture group, the learning effect in accuracy was significantly higher in the three mixture groups (the objective group, $F(1, 23) = 68.19$, $p < .001$; the visibility group, $F(1, 14) = 35.51$, $p < .001$; the confidence group, $F(1, 15) = 8.87$, $p = .009$).

The same method was used to compare priming and learning effects, by contrasting a full model that assumed two different *B* values and a restricted model that assumed the same *B* value. The priming effect was comparable to the learning effect in accuracy (relative to the visibility group, $p = .174$; to the confidence group, $p = .155$), but was significantly higher than the learning effect in ratings (relative to the visibility group: $F(1, 11) = 5.85$, $p = .034$; to the confidence group: $F(1, 12) = 18.21$, $p = .001$).

A statistical account of differing individual differences between priming and learning. Here we assess whether a statistical account might by itself explain why the priming effect appeared to be more robust than the learning effect. Specifically, in calculating the two effects, the number of trials was not equal: 140 trials each for the pre-mix and mix conditions (priming); 40 trials each for the first single block and the single block with peak performance (learning). This difference raises two questions, addressed in turn below.

First, could certain blocks show a much more robust priming effect than other blocks? Fig. S3 shows individual participants' accuracy and rating effects across the 14 blocks, as well as the mean and *SD*. The priming effect was fairly consistent across blocks: the mean accuracy effect ranged from 7.0% to 16.4% (*SD*, 18.4% to 25.8%); the mean rating effect, 0.58 to 0.84 (*SD*, 0.49 to 0.70). The priming effect did not statistically differ across blocks, either in accuracy ($F(13, 715) = 1.22$, $p = .260$, $\eta_p^2 = 0.02$) or in ratings ($F(13, 715) = 1.10$, $p = .359$, $\eta_p^2 = 0.02$), indicating a consistent priming effect across blocks.

Second, how many blocks of trials were needed to yield a robust priming effect? Fig. S3 presents accuracy and rating effects from individual participants as averaged across the first *n* blocks ($n = 1$ to 14). Inspecting the figure reveals that when more blocks are being averaged, the data points are more clustered together, more so for accuracy than for ratings. Specifically, in accuracy, after one block of trials, adding three more blocks each helped reduced standard deviation by 5.7% (Bartlett's test for equal variances, $T(13) = 181.9$, $p < .001$), 3.4% ($T(12) = 79.5$, $p < .001$), and 2.8% ($T(11) = 30.3$, $p = .001$). Importantly, after a total of 4 blocks (40 trials each condition), adding more blocks did not further reduce the variance ($T(10) = 7.9$, $p = .636$). In ratings, after 1 block (10 trials each condition), adding more blocks did not further reduce the variance ($T(13) = 9.4$, $p = .743$). In other words, to obtain a robust priming effect, 40 trials (4 blocks) per condition were sufficient for accuracy, and for ratings 10 trials (1 block) were sufficient.

Given that 40 trials (or 10 trials) and 140 trials did not differ in the variance in their priming effects, this finding suggests that more robust priming than learning was unlikely to be

due to more trials being averaged in priming than in learning. To confirm, we reanalyzed the priming effect using only the first 4 blocks (40 trials each condition), completely matching the trial number used in learning. Fig. S4a shows that, in priming, 80% of participants showed improvements in accuracy, and 98% of them also showed corresponding improvements in ratings, significantly higher than that in learning (Fig. 3a, X^2 (1, N = 84) = 13.95, $p < .001$). For replication, we also reanalyzed the learning effect, this time using the difference between the first single block and the last single block (instead of the peak performance block, as in Fig. 3a). Fig. S4a shows that, in learning, 52% of participants showed improvements in accuracy, and just 60% of them showed corresponding improvements in ratings—significantly lower than that in priming (Fig. S4a, X^2 (1, N = 72) = 15.28, $p < .001$). As before, it was not the case that objective and subjective measures were more dissociable in learning than in priming; if anything, Pearson's correlation between accuracy and rating effects was numerically higher for learning (r = .46) than for priming (r = .30), although the difference was not significant (z = 1.00, p = .317).

Finally, when looking at accuracy effects based on *positive rating* effects, the pattern was similar. As Fig. S5 shows, for learning, 46% of participants showed improvements in ratings, and only 58% of them showed corresponding improvements in accuracy. For priming, 96% of participants showed improvements in ratings, and 85% of them also showed corresponding improvements in accuracy, significantly higher than that in learning (X^2 (1, N = 80) = 5.89, p = .015).

Three Supplementary Tables follow:

Table S1. Model fitting and parameter estimation for learning in single blocks

	r^2	*A*	*B*	*T*	*p* value
**Accuracy**					
Non-mixture group	0.35 (0.21)	58.8 (1.3)	**2.4** (3.1)	6.5 (5.6)	= .005
Objective group	0.82 (0.14)	58.3 (1.8)	**13.6** (3.5)	0.9 (0.4)	< .001
Visibility group	0.98 (0.10)	64.6 (2.6)	**10.6** (3.9)	0.9 (1.5)	= .004
Confidence group	0.88 (0.20)	62.7 (2.6)	**7.6** (4.0)	1.6 (3.4)	= .005
**Ratings**					
Visibility group	0.95 (0.23)	1.63 (0.07)	**0.14** (0.20)	1.4 (4.9)	= .013
Confidence group	0.60 (0.25)	1.63 (0.07)	**0.12** (0.11)	0.9 (3.4)	= .037

Note: Block-by-block data from single blocks were fitted to the learning model, $A + B\,(1 - e^{-(t-1)/T})$, with 3 parameters: *A*, initial performance; *B*, maximum amount of learning; *T*, the time constant for learning, which determines the shape of the curve (i.e., the learning rate). For the non-mixture group and the objective group, the data included 14 block pairs; for the visibility and confidence groups, because of fatigue, the data included up to the block pair with the highest identification accuracy (block pairs 5 and 6, respectively). The parameter *A* was fitted using the performance level from block pair 1 ($t = 1$). Standard deviations are within parentheses, calculated from 1000 repetitions of bootstrap resampling.

Table S2. Model fitting and parameter estimation for priming and its decay

	r^2	*A*	*B*	*T1*	*T2*	*p* value
**Accuracy**						
Objective group	0.54 (0.12)	71.3 (2.8)	**6.6** (1.9)	0.8 (1.6)	10.1 (3.0)	< .001
Visibility group	0.74 (0.09)	70.7 (2.8)	**9.4** (1.7)	1.2 (0.5)	8.8 (3.4)	< .001
Confidence group	0.77 (0.05)	66.8 (2.3)	**14.0** (1.4)	1.1 (0.8)	9.7 (2.2)	< .001
All 3 groups	0.84 (0.04)	69.6 (1.5)	**10.0** (1.0)	1.1 (0.4)	9.6 (1.4)	< .001
**Ratings**						
Visibility group	0.97 (0.02)	1.72 (0.09)	**0.67** (0.08)	1.2 (0.2)	4.8 (0.7)	< .001
Confidence group	0.97 (0.03)	1.72 (0.09)	**0.86** (0.08)	0.7 (0.1)	4.5 (0.8)	< .001
All 2 groups	0.98 (0.01)	1.72 (0.06)	**0.76** (0.05)	0.9 (0.1)	4.6 (0.6)	< .001

Note: Trial-by-trial data were fitted to the priming–decay model, $A + B\,(1 - e^{-(t-1)/T1})$, when $t < 11$ (i.e., for pre-mix and mix trials); $A + B \times e^{-(t-11)/T2}$, when $t \geq 11$ (i.e., for post-mix and single-block trials). There are 4 parameters: *A*, initial performance; *B*, maximum amount of priming; *T1*, time constant of priming, which determines the rising rate; *T2*, time constant of decay, which determines the decline rate. The *p* value was obtained by comparing the model with the no priming (decay) model (i.e., $B = 0$). Data included 61 data points: the first data point ($t = 1$) was the mean performance of the 10 hard trials in the pre-mix segment (baseline); the rest of the data points were the subsequent hard trials across each pair of mixed and single blocks. The parameter *A* was fitted using the first data point. Standard deviations are within parentheses, calculated from 1000 repetitions of bootstrap resampling.

Table S3. Model fitting and parameter estimation for learning in mixed blocks

	r^2	*A*	*B*	*T*	*p* value
**Accuracy**					
Premix	0.61 (0.16)	61.0 (1.7)	**9.6** (2.5)	0.9 (0.2)	< .001
Mix	0.69 (0.20)	73.1 (2.1)	**6.1** (2.2)	0.6 (1.1)	< .001
Postmix	0.07 (0.11)	74.0 (2.1)	**1.4** (2.2)	0.1 (5.2)	= .020
**Ratings**					
Premix	–	–	–	–	= .939
Mix	0.49 (0.23)	2.30 (0.07)	**0.15** (0.08)	0.3 (2.5)	< .001
Postmix	0.17 (0.18)	1.93 (0.07)	**0.09** (0.10)	0.1 (5.1)	< .001

Note: Block-by-block data from mixed blocks—separated into premix, mix, and postmix segments—were fitted to the learning model, $A + B\,(1 - e^{-(t-1)/T})$, with 3 parameters: *A*, initial performance; *B*, maximum amount of learning; *T*, the time constant for learning, which determines the shape of the curve (i.e., the learning rate). The *p* value was obtained by comparing the learning model with the no-learning model (i.e., $B = 0$). The accuracy data were pooled from the objective, visibility, and confidence groups; the rating data, the visibility and confidence groups. The parameter *A* was fitted using the performance level from the first mixed block ($t = 1$). Standard deviations are within parentheses, calculated from 1000 repetitions of bootstrap resampling.

Six Supplementary Figures follow:

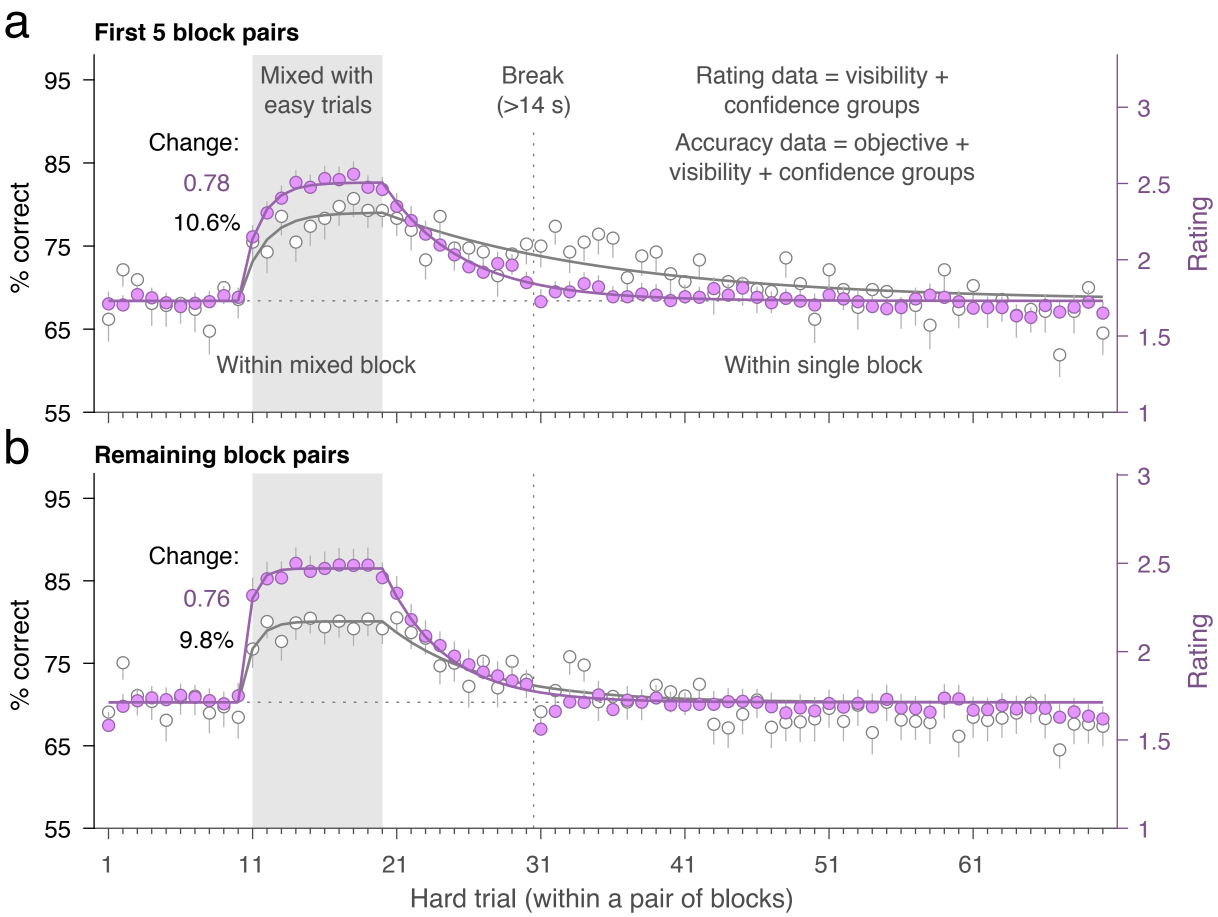

Figure S1. Trial-by-trial transient effects in early and late stages of the session. (**a**) The trial-by-trial transient effect was evident in the first 5 block pairs, where performance in the single blocks rapidly improved. (**b**) The transient effect persisted for the remaining blocks, where performance in the single blocks stabilized. Note the slightly different scales for ratings across the two panels. Numbers are effect sizes in perceptual gain (based on the parameter *B* in the priming–decay model). Error bars (one-sided) are standard errors of the mean.

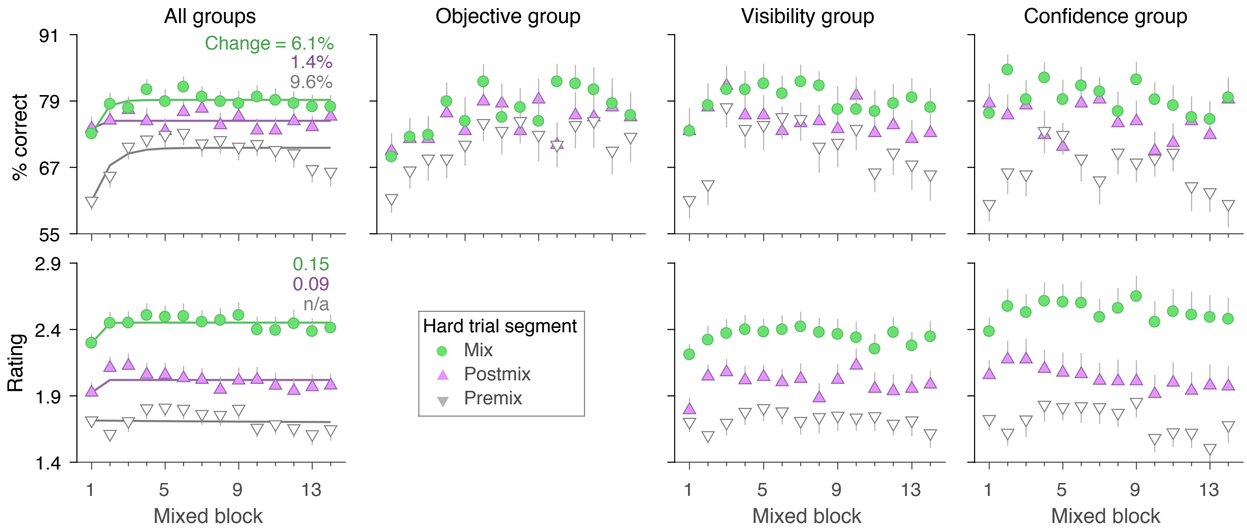

Figure S2. Fast learning in mixed blocks. Learning effect (*B*) in accuracy manifested primarily in the premix and mix segments, with comparable magnitude to that in the single blocks (Fig. 2a) and to the priming effect (Fig. 2b and Fig. S1). The rating effect was also comparable to that in the single blocks (Fig. 2a)—but both much smaller than the priming effect in ratings (Fig. 2b and Fig. S1). Each data point represents the average of 10 hard trials. The connecting lines were fitted based on an exponential learning model (see Table S3). Numbers are effect sizes in perceptual gain (based on the parameter *B* in the learning model). Error bars (one-sided) are standard errors of the mean.

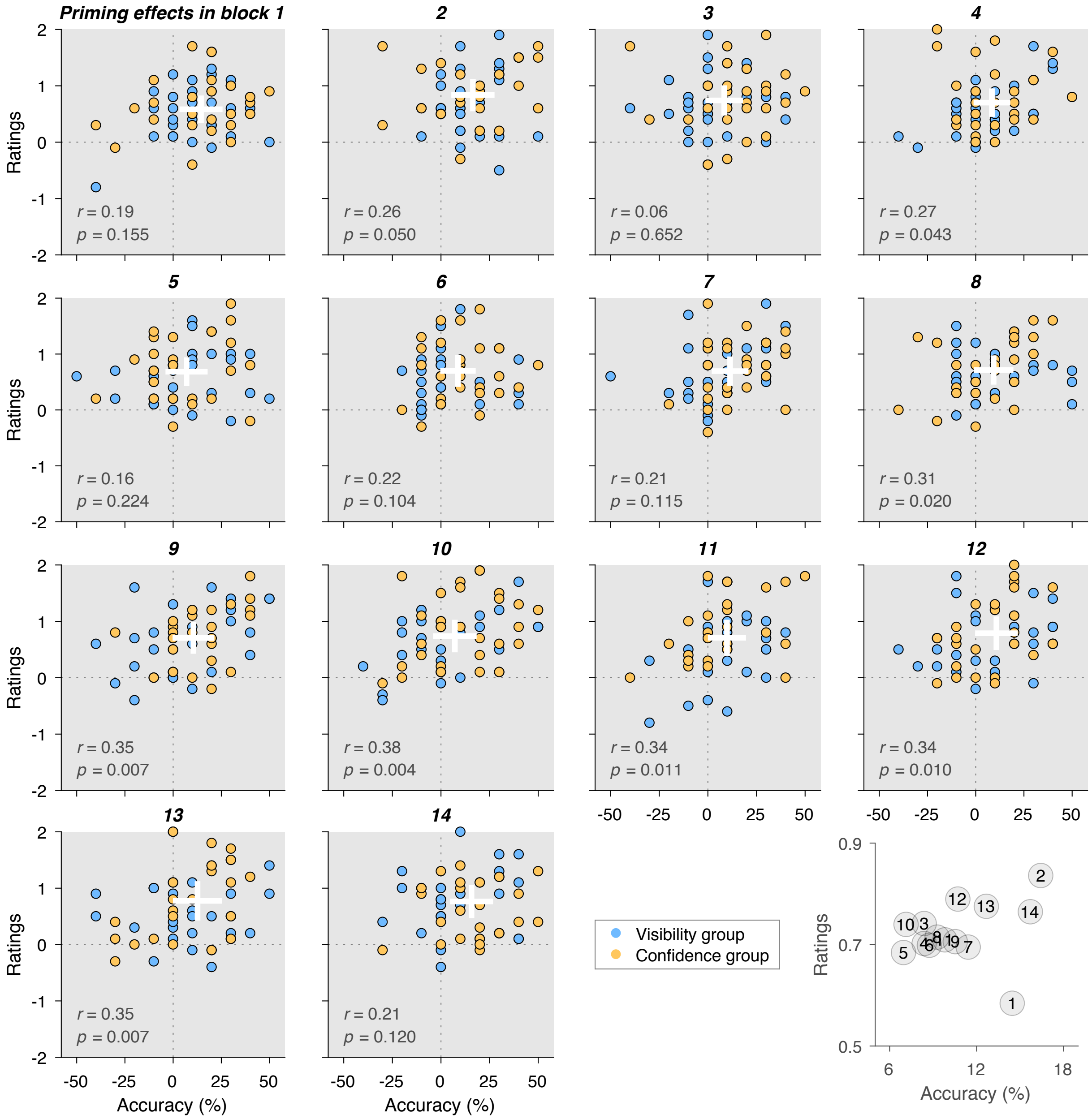

Figure S3. Consistent priming effect across blocks. In both accuracy and ratings, the priming effect was comparable across the 14 blocks, with similar means and standard deviations. Each dot on the graph represents an individual participant; center of the white bar, the mean; full length of the white bar, a standard deviation. Bottom left in each graph shows Pearson's correlation between accuracy and rating effects. The mean effects in each block are aggregated in the insert at the bottom right.

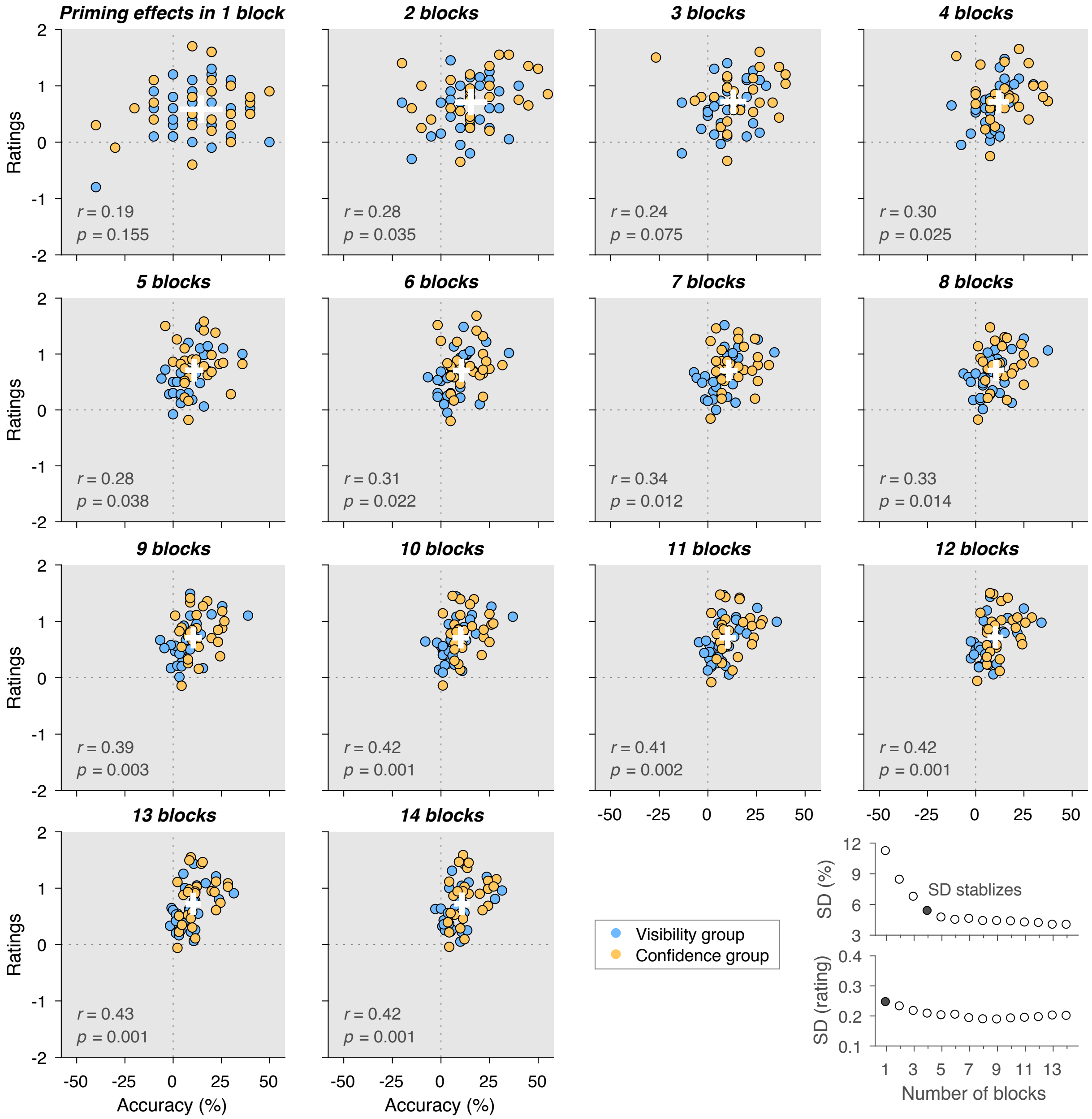

Figure S4. Robust priming effects from 40 trials (4 blocks) for accuracy and from 10 trials (1 block) for ratings. The variance in the accuracy effect was greatly reduced after up to 4 blocks of data were included, but barely changed after just 1 block for the rating effect (see main text above for statistics). Each dot on the graph represents an individual participant; center of the white bar, the mean; full length of the white bar, a standard deviation. Bottom left in each graph shows Pearson's correlation between accuracy and rating effects. The standard deviations from the 14 small graphs are aggregated in the insert at the bottom right, separately for accuracy and ratings.

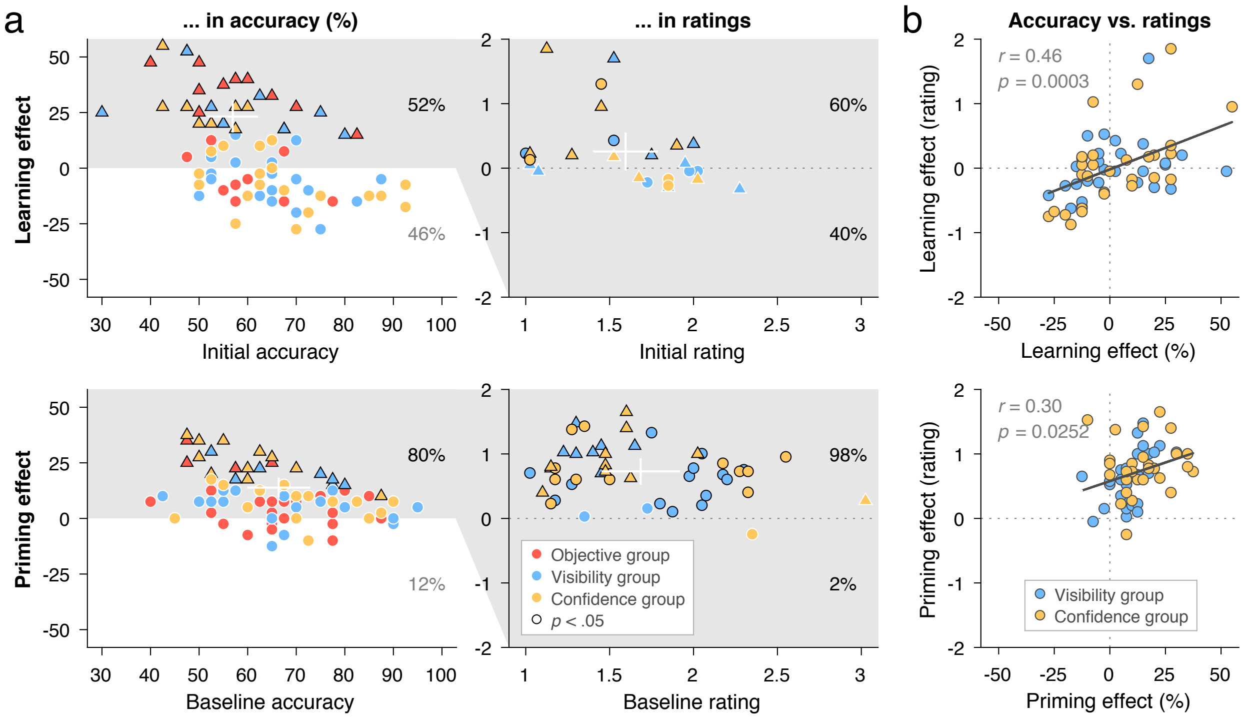

Figure S5. Distinct contributions of learning and priming to rapid perceptual enhancement in hard trials. (**a**) Learning was calculated by the performance difference between the first single block and the last single block (instead of the peak performance block, as in Fig. 4). The learning effect was positive in terms of *accuracy* in 52% of participants (left), and only a subset of them showed a corresponding *subjective* effect (right). For the priming effect, four blocks (instead of 14 blocks, as in Fig. 4) were included in the calculation, with 40 trials each for the mix and premix hard trials. The priming effect was positive in terms of *accuracy* in 80% of participants (left), and most of them showed a corresponding *subjective* effect (right). Percentages represent the proportions of participants showing positive or negative effects. Each dot or triangle represents an individual participant (triangle denotes statistically significant accuracy effect at the individual level, $p < .05$, one-tailed); black contour denotes statistically significant effect at the individual level, $p < .05$ (white contour, non-significant effect); center of the white bar, the mean of the individuals within the highlighted (gray) region; full length of the white bar, a standard deviation. (**b**) Accuracy effects and rating effects were positively correlated with each other; the magnitude of correlations did not statistically differ in learning and priming.

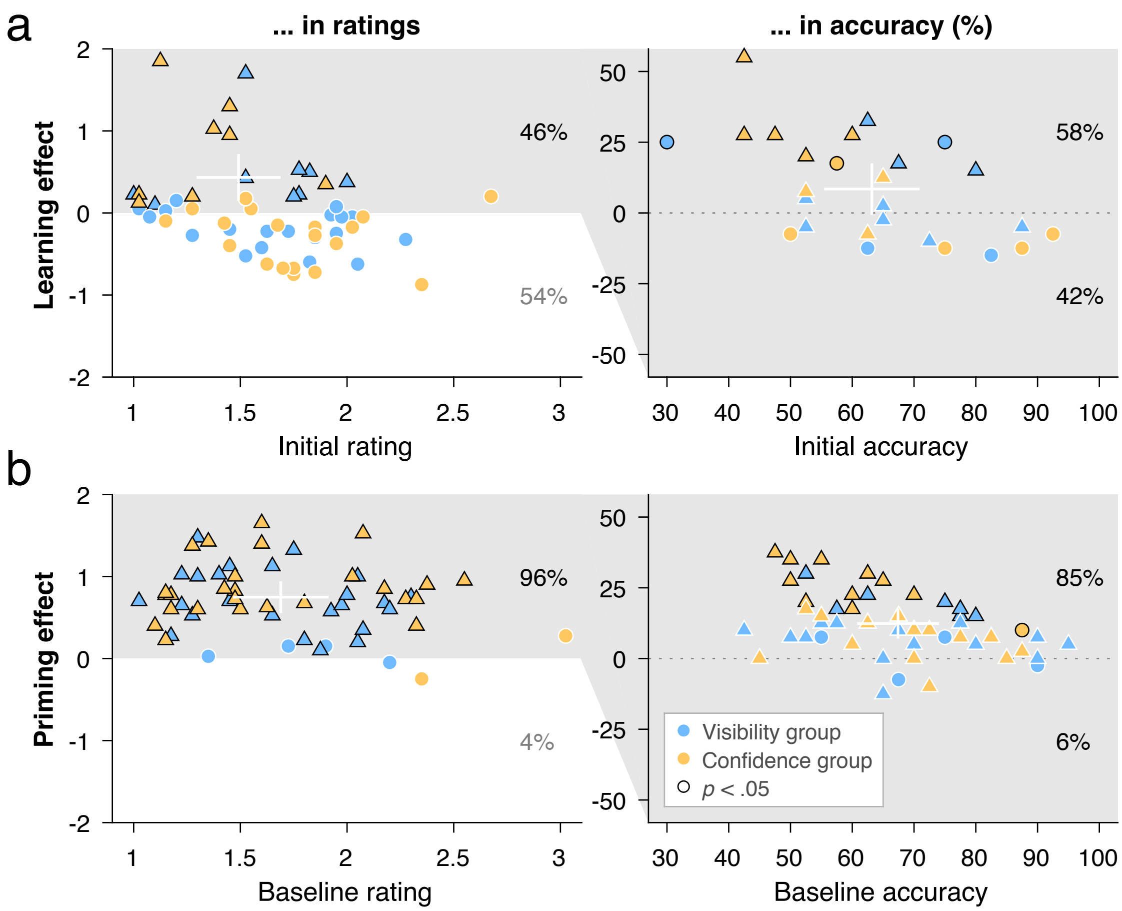

Figure S6. Distinct contributions of learning and priming to rapid perceptual enhancement in hard trials, as revealed by conditioning accuracy effects on positive rating effects. (a) Learning was positive in terms of *ratings* in 46% of participants (left), and only a subset of them showed a corresponding *accuracy* effect (right). (**a**) Priming was positive in terms of *ratings* in 96% of participants (left), and most of them showed a corresponding *accuracy* effect (right). Note that with the same data set the order of conditioning (testing accuracy effects based on positive rating effects) was the reverse of Fig. S3. Percentages represent the proportions of participants showing positive or negative effects. Each dot or triangle represents an individual participant (triangle denotes statistically significant rating effect at the individual level, $p < .05$, one-tailed); black contour denotes statistically significant effect at the individual level, $p < .05$ (white contour, non-significant effect); center of the white bar, the mean of the individuals within the highlighted (gray) region; full length of the white bar, a standard deviation.
